# Enhanced knowledge of spontaneous reporting with structured educational programs in Korean community pharmacists: a cross-sectional study

**DOI:** 10.1186/s12909-017-0933-0

**Published:** 2017-05-30

**Authors:** Yun Mi Yu, Euni Lee

**Affiliations:** 1 College of Pharmacy & Research Institute of Pharmaceutical Sciences, Seoul National University, 103 Daehak-ro, Jongno-gu, Seoul, 03080 South Korea; 2 College of Pharmacy & Research Institute of Pharmaceutical Sciences, Continuing Education Center for Advanced Pharmacy, Seoul National University, 1 Gwanak-ro, Gwanak-gu, Seoul, 08826 South Korea

**Keywords:** Knowledge, Continuing education, Spontaneous reporting, Adverse drug reaction reporting system, Pharmacist, Under-reporting

## Abstract

**Background:**

While spontaneous reporting (SR) is one of the important public health activities for community pharmacists to guard patients’ safety, very few studies examined educational activities and its effects on knowledge about the SR system in Korea. This study described the association between knowledge of SR and educational activities targeting community pharmacists in Korea.

**Methods:**

Self-administered questionnaires were collected between September 1, 2014 and November 25, 2014. The questionnaires addressed sources of SR knowledge (structured educational programs, personal access to educational resources, and information by social network services) and knowledge about the Regional Pharmacovigilance Center designated for community pharmacists, the legal responsibility clause on the serious event reporting, and the reportable items. The association between the knowledge of SR and the educational activities was evaluated using analysis of variance or chi-squared tests.

**Results:**

Overall, 766 questionnaires demonstrated that mean age and length of career in community pharmacies was 45.7 years and 15.9 years, respectively. A structured educational program was used in 63.1% of the participants followed by a personal access to educational resources (56.3%). An educational program offered by the Korean Pharmaceutical Association was the most frequently mentioned program (56.8%), and no regional disparity in the program between the metropolitan and rural areas was observed. Pharmacists who had personal access to educational resources identified SR knowledge contents less correctly than those who used a structured educational program or both (*p* < 0.01). In general, pharmacists’ knowledge on reportable items was significantly lower with non-prescription drugs, nutritional supplements, and personal hygiene products as compared to their knowledge on prescription drugs, regardless of the type of education (*p* < 0.01).

**Conclusions:**

Knowledge regarding SR was more likely to increase when a structured educational program was used alone or in combination with other educational methods. Knowledge on reportable items should be reinforced during the continuing education process.

## Background

Spontaneous reporting (SR) of adverse drug reactions (ADRs) plays a critical role in identifying drug safety signals and improving quality of patient care by the early detection of new or rare ADRs [1]. The safety information listed in the approved package inserts were mostly based on preapproval studies that included a relatively small number of patients and a short duration of drug exposure [2]. The successful operation of the SR system and utility of the safety signals for policy implementation are important for including safety updates in the package insert, and these activities are heavily dependent on accurate, timely, and vigilant reports made by the reporters.

While improved reporting rates have been published from a few countries [3, 4], a systematic review including 37 studies showed under-reporting rates of higher than 90%; the under-reporting was identified as one of the major hurdles in improving the pharmacovigilance outcomes [5]. With concerted efforts made by the government and healthcare professionals in Korea, increased SR activities have been documented since the establishment of the Korea Institute of Drug Safety and Risk Management (KIDS), the government agency responsible for the improvement of pharmacovigilance and comprehensive management of the Regional Pharmacovigilance Centers (RPVCs) across the country [6]. In addition, more SR cases from community pharmacists were submitted to KIDS after the active involvement of the Korean Pharmaceutical Association (KPA) designated as a RPVC to collect the SR by community pharmacists nationwide in Korea [3], which could be considered as a successful collaboration between the government and the professional organization representing community pharmacists in Korea. Although the overall reporting rate has increased, there is still room to improve in Korea as the increase was relatively lower than that from other countries, e.g., in the Netherlands, Spain, or Portugal [4].

Several published studies showed that the knowledge of health care professionals was a major predictive factor for under-reporting in comparison with the personal and professional characteristics of physicians and pharmacists [7–10]. Educational programs and educational resources have been frequently recommended as the method to increase knowledge of SR [5, 7–9, 11]. While strategies like educational programs or various methodologic approaches are needed to improve under-reporting in Korea, very few studies examined educational activities and its effects on knowledge about the SR system and reportable events by education types and resources in Korea [12, 13]. Also, data on SR related educational activities including Korean community pharmacists are extremely limited. Therefore, this study aimed to describe types of educational activities on the SR system and to evaluate the association between knowledge of SR and educational activities targeting community pharmacists.

## Methods

### Survey Instrument

The survey instrument contained 10 questions on the types of educational activities, the knowledge of SR, and demographic information such as age, gender, length of career in community pharmacy and the location of the pharmacy. The geographic location included 7 metropolitans and 9 provinces representing all administrative districts in Korea. The question about the types of educational activities included multiple selection options; structured and formal educational programs, information leaflet, media such as Internet sites for pharmaceutical news or KIDS homepage, and information by co-worker or social network services. The question about the types of structured and formal educational programs also included multiple selection options; the education by KPA, the education by other professional organization, online education, and entry-level pharmacy program for college students.

In this study, knowledge of SR was measured using a composite score from 3 survey items with each correct answer counting as 1 point: having knowledge on the designation of KPA as one of the Regional Pharmacovigilance Centers to collect ADRs (1 point), knowledge on the legal responsibility clause on the serious event reporting (1 point), and knowledge on the reportable items (1 point). For the knowledge on reportable items, the participants should select all four options including prescription drugs, non-prescription drugs, nutritional supplements, and personal hygiene products to be assigned to 1 point.

### Sample Size

Based on the findings from a published study by Irujo and colleagues indicating an 18% difference of knowledge score between two groups with different levels of structured education [14], a sample size of 720 subjects (240 subjects per group) in three-group analysis was calculated as adequate to detect a 15% frequency difference with 80% power and 5% α-error (Epi Info™ 7.1.5, Centers for Disease Prevention and Control, Atlanta, GA).

### Survey distribution

A cross-sectional survey study was conducted using a self-administered questionnaire with a nationwide convenience sample of community pharmacists in all 16 administrative districts in Korea. A detailed description of the survey development, subject recruitment, and response rate was described in previous literature [15]. Briefly, the questionnaire using the mixed theoretical model [16] of the knowledge-attitude-practices model [17, 18] and the theory of satisfaction of needs [19] was developed. The survey was distributed either online using a pharmacy billing program called the PM2000 that is used by the majority of community pharmacies in Korea or by a paper-based method from two national-level conferences targeting pharmacists who did not prefer the online platform or used a different billing program between September 1, 2014 and November 25, 2014. The study population included pharmacists who reported their knowledge on the presence of SR system in Korea and identified one or more educational activities.

All survey participants provided their written informed consent to participate in this study while ensuring confidentiality to meet our ethical standards. A full ethical review was made for all procedures following the protocol approved by the Institutional Review Board (IRB) of Seoul National University, and the study was approved by the IRB (IRB No. E1410/001–011).

### Data analysis

Pharmacists were classified into three groups by types of educational activities: 1) structured education, 2) personally accessed educational resource, or 3) combination. The structured education group consisted with the pharmacists who had knowledge regarding SR through only the structured and formal educational programs. The personally accessed educational resource group consisted of pharmacists who acquired their knowledge regarding SR through the personally accessed educational resources, such as an information leaflet, media, or information by co-worker or social network services. The combination group included pharmacists who received both structured education and utilized educational resources by individual access. The location of community pharmacies was divided into metropolitan area (7 major cities) and rural area (9 provinces) in data analysis representing all 16 administrative districts in Korea.

The demographic and clinical characteristics of the three groups were presented with descriptive statistics, such as mean, standard deviation (SD), frequency, and percentage. The continuous and categorical variables between the three groups were compared using an analysis of variance (ANOVA) test and chi-squared tests, respectively. Statistical differences of demographic variables and pharmacists’ knowledge on the individual items and the scores for the 3 items within each type of educational approach were assessed. Statistical differences of individual educational activities according to the location of the community pharmacy between metropolitan area and rural area were also compared using chi-squared tests. The significance level was set at *p* < 0.05. For the post hoc analysis, the Bonferroni correction was employed. Data analysis was performed using SPSS version 22.0 (SPSS Inc., Chicago, IL).

## Results

From September 1, 2014 to November 25, 2014, a total of 1004 of the 1315 invited community pharmacists participated in this study and the response rate was 76.3% [15]. Two hundred thirty participants answering that they did not know the existence of SR system were excluded. Of all collected 774 questionnaires, the study included 766 questionnaires, i.e., 450 online and 316 for the paper-based survey; eight questionnaires that did not include the source of the knowledge on SR were excluded.

### Characteristics of study population

The mean (± SD) age of the study population was 45.7 (± 10.7) years, and females comprised 57.4% of the study population (Table 1). The mean (± SD) career length in community pharmacies was 15.9 (± 10.5) years. For the sources to obtain knowledge regarding SR, structured educational programs were used by 483 pharmacists (63.1%), while personally accessed educational resources were used by 431 pharmacists (56.3%). Education by the KPA (435 pharmacists, 56.8%) was the most frequent in the structured educational programs, and information leaflets (175 pharmacists, 22.8%) were the most prevalent in the personally accessed educational resources.

**Table 1:** Population demographics (*n* = 766)

Characteristics	Value
Age, mean ± SD (years)	45.7 ± 10.7
Sex, *n* (%)	
Male	320 (41.8)
Female	440 (57.4)
Unknown	6 (0.8)
Career in community pharmacy, mean ± SD (years)	15.9 ± 10.5
Location of the pharmacy, *n* (%)	
Metropolitan area	473 (61.7)
Rural area	292 (38.1)
Unknown	1 (0.1)
Types of educational activities^a^, *n* (%)	
*Structured educational programs*	
Education by KPA	435 (56.8)
Educational activities by other professional organization	73 (9.5)
Online education	55 (7.2)
Entry-level pharmacy program	40 (5.2)
*Personally accessed educational resources*	
Information leaflet	175 (22.8)
Media	149 (19.5)
Information by co-worker	134 (17.5)
Information by social network services	50 (6.5)

*Abbreviations KPA* Korean Pharmaceutical Association

^a^Each respondent could select more than one option

### Characteristics according to types of educational activities

The structured education group, the personally accessed educational resource group, and the combination group comprised 43.7% (335 pharmacists), 37.0% (283 pharmacists), and 19.3% (148 pharmacists) of the population, respectively (Table 2). The mean age and length of career in community pharmacies were significantly higher in the combination group than the resource or education groups (48.1 years vs. 44.4 or 45.0 years for mean age; 18.2 years vs. 15.6 or 15.2 years for career in community phasrmacy; *p* < 0.05, Table 2). There was no significant difference in gender and location of community pharmacies among the three groups.

**Table 2:** Characteristics of the pharmacists and knowledge contents by types of educational activities

Characteristics	Personally accessed educational resource group^a^ (*n* = 283)	Structured education group^b^ (*n* = 335)	Combination group^c^ (*n* = 148)	*p* value^*^	Post hoc^†^
Characteristics					
Age, mean (SD)	44.4 (11.0)	45.0 (10.4)	48.1 (10.7)	0.012	c > a,b
Career in community pharmacy, mean (SD)	15.6 (10.6)	15.2 (10.4)	18.2 (10.4)	0.014	c > a,b
Male, *n* (%)	115 (40.8)	135 (40.8)	70 (47.6)	0.321	
Metropolitan area, *n* (%)	165 (58.5)	211 (63.0)	97 (65.5)	0.306	
SR Knowledge contents					
Knowledge of RPVC-KPA^‡^, *n* (%)	202 (71.9)	272 (81.7)	122 (84.1)	0.002	b,c > a
Knowledge on related laws^‡^, *n* (%)	74 (26.3)	138 (41.3)	69 (46.6)	<0.001	b,c > a
Knowledge on reportable items^‡^, *n* (%)	91 (32.2)	135 (40.5)	59 (39.9)	0.078	

*Abbreviations ADR* Adverse drug reaction, *SR* spontaneous reporting, *RPVC-KPA* Regional Pharmacovigilance Center at the Korean Pharmaceutical Association

^a^Personally accessed educational resource group (*n* = 283)

^b^Structured education group (*n* = 335)

^c^Combination group (*n* = 148)

^*^Analysis of variance test or chi-squared test among the three groups

^†^Bonferroni correction with student’s t-test or chi-squared test

^‡^The number of pharmacists who reported their knowledge on each knowledge content area

While there was no regional difference between metropolitan area and rural area in the proportion of the education by KPA, online, or education from the entry-level pharmacy program or each of the personally accessed educational resources, the proportion of education by other professional organizations was significantly different between the metropolitan and rural areas (12.1% vs. 5.5%, *p* = 0.003).

### Knowledge according to types of educational activities

**Fig. 1:**
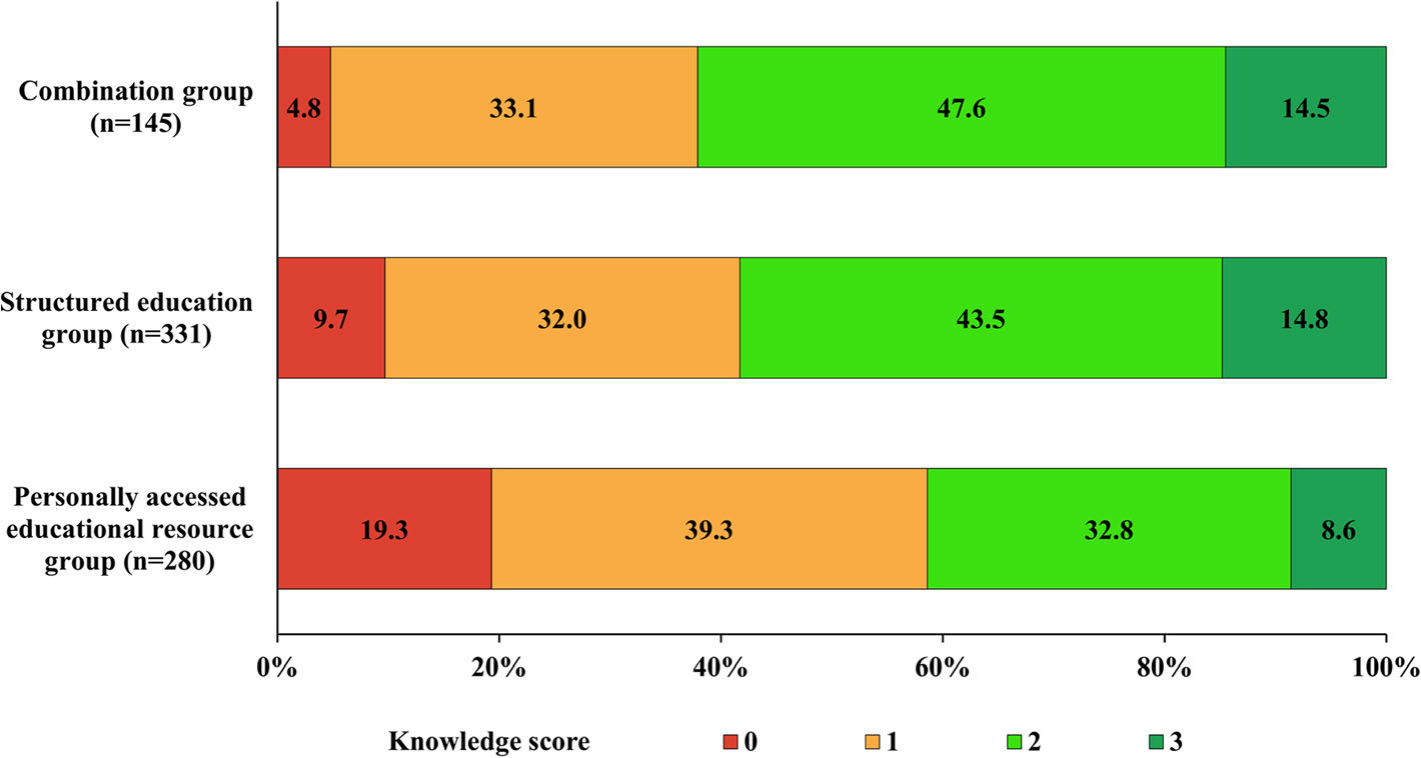
Comparison of the knowledge score by the type of educational activities (*n* = 756). The knowledge was measured in scores on 3 following survey items with each correct answer being 1 point; 1) knowledge of Regional Pharmacovigilance Center-Korean Pharmaceutical Association, 2) legal responsibilities related to the reporting of serious events, and 3) reportable items. *Red bar*: zero point; *Orange bar*: one point; *Light green bar*: two points; *Dark green bar*: three points

Comparing the numbers of correctly identified content areas on SR knowledge, knowledge was significantly lower in the personally accessed educational resource group than the other groups (*p* < 0.01, Fig. 1). Nearly one-fifth (19.3%, 54 pharmacists) of the personally accessed educational resource group had no knowledge, and only 8.6% (24 pharmacists) had correct knowledge regarding all three content areas. In particular, the personally accessed educational resource group showed significantly lower knowledge on the Regional Pharmacovigilance Center-KPA and SR-related laws compared to the other groups (*p* < 0.05, Table 2).

Considering knowledge on reportable items, knowledge on prescription drugs was similarly high (above 97.0%), and their knowledge was significantly decreased with non-prescription drugs, nutritional supplements, and personal hygiene products as compared to their knowledge on prescription drugs in all groups (*p* < 0.01, Table 3). When their knowledge was compared between different types of educational activities, knowledge on nonprescription drugs was significantly lower in the personally accessed educational resource group compared with that of the structured education group (76.7% vs. 87.7%, *p* < 0.001, Table 3).

**Table 3:** Knowledge on reportable items by types of educational activities^*§^

Reportable items	Personally accessed educational resource group^a^ (*n* = 283)	Structured education group^b^ (*n* = 335)	Combination group^c^ (*n* = 148)	*p* value^†^	Post hoc^‡^
Prescription drugs, *n* (%)	276 (97.5)	324 (97.3)	147 (99.3)	0.357	
Non-prescription drugs, *n* (%)	217 (76.7)	292 (87.7)	127 (85.8)	0.001	b > a
Nutritional supplements, *n* (%)	168 (59.4)	215 (64.6)	91 (61.5)	0.410	
Personal hygiene products, *n* (%)	99 (35.0)	146 (43.8)	65 (43.9)	0.054	

^a^Personally accessed educational resource group (*n* = 283)

^b^Structured education group (*n* = 335)

^c^Combination group (*n* = 148)

^*^The number of pharmacists who correctly identified each reportable item

^†^Chi-squared test between the groups

^‡^Bonferroni correction with chi-squared test

^§^Statistical differences within each group was tested by chi-squared test; *p* < 0.001 for personally accessed educational resource group or structured education group, *p* < 0.01 for combination group

## Discussion

To the best of our knowledge, this is the first nationwide survey to study the effect of educational activities on knowledge of SR in Korea. While pharmacists’ knowledge was a major determinant in under-reporting and educational activities have increased knowledge of SR [7–9], very few studies have evaluated knowledge by the type of educational activities in Korea. Our findings showed that educational resources alone were less associated with knowledge of SR than structured educational programs. Although a recent study corroborated our findings with printed educational materials showing a beneficial, yet small, effect on professional practice outcomes [20], Bracchi and colleagues demonstrated ADR reports were increased using an educational bulletin linked to education credits in a distance learning program [21].

Knowledge of SR, in our study, showed a significant relationship with the structured educational program, which consisted of several sessions regarding to the importance and the advantages of ADR reporting, the disadvantages of under-reporting, and the method of SR. This is consistent with published studies including pharmacists and nurses, which have showed that the structured educational intervention improved the lack of knowledge of SR [12, 13]. Findings from our study on no regional disparity in the education by KPA between the metropolitan and rural areas could be partially explained by the administrative strategy of the national-level pharmacy association in closely working with local KPA branches through mandating continuing education (CE) requirements on medication safety and spontaneous reporting.

The CE is a leading educational program for health professionals and offers several influential characteristics of persistency and consistency, which would contribute to the desired educational outcomes [22, 23]. Most U.S. State Boards of Pharmacy mandate 30 or more hours of CE credits for licensure renewal mandating their CE credits on the medical error or safety related to SR [24]. Great Britain, Singapore, and Canada incorporated a revalidation system with optional education regarding ADR reporting [25–27]. In Korea, mandatory CE to meet the pharmacist licensure requirements is 6 h per year and education about patient safety including SR is also optional [28]. The Ministry of Health and Welfare, the agency in charge of issuing licensures for health professionals in Korea, have delegated the management of CE for pharmacists to KPA [28]. Considering the advantage of SR education by KPA as an important portal for pharmacovigilance activities, we believe that the drug safety education should be designated as a required CE activity and the yearly required credit of 6 h may not sufficiently cover educational contents on drug safety for practicing pharmacists in Korea.

In our study, the fact that one in five pharmacists (i.e., 19.5%) utilizes media materials as personally accessed educational resources can have important implications. As the access to the Internet became democratized in the global community, enhanced access to more credible drug safety information sites on the Internet can be a potential solution for improving pharmacists’ knowledge [29]. Especially, persistent efforts advancing the health information technology are apparent in Korea such that the Korean Pharmaceutical Information Center has provided a one-click information link to the pharmacy’s billing program used by all community pharmacies under the national health insurance system network.

Findings from our study indicated that regardless of the type of educational activities, the knowledge on reportable items significantly decreased with non-prescription drugs, nutritional supplements, and personal hygiene products as compared to knowledge on prescription drugs. As community pharmacists can serve as the primary healthcare provider in helping patients on self-care with these products [6, 30], knowledge regarding these reportable items is extremely important for community pharmacists, and the suboptimal level of knowledge as demonstrated in our study can be concerning; therefore, strategies to improve knowledge regarding reportable items are needed.

This study has some limitations. First, we relied on voluntary participation and a convenience sample of pharmacists in this survey. This has the potential for selection bias with limited generalizability. However, this study showed good response rate of over 75%, which may allow overcoming the concerns about the limited generalizability. Second, concerning the self-report study, this study has the potential for social desirability bias and recall bias. Third, other types of educational resources that are not explained in the survey can be available. Although our study did not explore resources, we focused on the most prevalently used educational activities in Korea.

## Conclusions

In conclusion, the findings from our study show that more than half of community pharmacists obtained SR information through the structured education by KPA, and the education by KPA was not different between the metropolitan and rural areas. Knowledge of SR was more likely to increase in the structured education or the combination of structured education and personally accessed educational resources. As the knowledge of the reportable items on non-prescription drugs, nutritional supplements, and personal hygiene products was lower than their knowledge on prescription drugs, more focused efforts that reinforce the educational contents are needed to improve the drug safety process targeting community pharmacists.

## Abbreviations

ADR: Adverse drug reaction
ANOVA: Analysis of variance
CE: Continuing education
IRB: Institutional Review Board
KIDS: Korea Institute of Drug Safety and Risk Management
KPA: Korean Pharmaceutical Association
RPVC: Regional Pharmacovigilance Center
RPVC-KPA: Regional Pharmacovigilance Center at the Korean Pharmaceutical Association
SD: Standard deviation
SR: Spontaneous reporting
